# Differences in circulating γδ T cells in patients with primary colon cancer and relation with prognostic factors

**DOI:** 10.1371/journal.pone.0243545

**Published:** 2020-12-16

**Authors:** Juan Carlos Andreu-Ballester, Lorena Galindo-Regal, Julia Hidalgo-Coloma, Carmen Cuéllar, Carlos García-Ballesteros, Carolina Hurtado, Natalia Uribe, María del Carmen Martín, Ana Isabel Jiménez, Francisca López-Chuliá, Antonio Llombart-Cussac

**Affiliations:** 1 Research Department, Arnau de Vilanova University Hospital, València, Spain; 2 Hematology Department, Arnau de Vilanova University Hospital, València, Spain; 3 Department of Medical Oncology, Arnau de Vilanova University Hospital, València, Spain; 4 Faculty of Pharmacy, Department of Microbiology and Parasitology, Complutense University, Madrid, Spain; 5 Faculty of Pharmacy, Laboratory of Parasitology, University San Pablo CEU, Madrid, Spain; 6 Department of General and Digestive Surgery, Arnau de Vilanova University Hospital, València, Spain; 7 Pathology Department, Arnau de Vilanova University Hospital, València, Spain; Sapporo Medical University School of Medicine, JAPAN

## Abstract

Downregulation of the T cell system has been proposed as a mechanism to block immunity in colonic cancer (CC). However, little has been studied about circulating αβ and γδ T cells and their immunological status in newly diagnosed patients. The aim of this study was to characterize the αβ and γδ T cell subsets in peripheral blood of patients with CC matched with healthy volunteers. In this prospective case-control study, blood samples were obtained from 96 patients with newly diagnosed treatment-naïve infiltrating colonic adenocarcinoma and 48 healthy volunteers. Pathological report at surgery was obtained from all CC patients. A significant decrease in CD3+ γδ T cells and CD3+CD8+ γδ T cells (p<0.001) were observed in CC patients. Apoptosis was significantly increased in all conventional and both αβ and γδ T cell subsets in patients with CC vs healthy subjects. γδ T cells were decreased in peripheral blood of patients with microscopic infiltration in tissues, history of cancer and synchronous colon cancer (p < 0.05). IFN-γ was significantly reduced in CC patients compared to controls. Cytotoxic effector γδ T cells TEMRA (CD8 and CD56) are the proportionally most abundant T cells in peripheral blood of CC patients. Patients with CC present a deep downregulation in the systemic T-cell immunity. These variations are evident through all tumor stages and suggest that a deficiency in γδ T cell populations could be preventing control of tumor progression. This fact prove the role of immunomodulation on CC carcinogenesis.

## 1 Introduction

Colorectal cancer (CRC) is the fourth most common cancer and the third cause of cancer-related death worldwide [1]. The carcinogenic process requires between five to ten years, a polyp which progresses to adenoma, dysplasia, and finally to carcinoma, with accumulation of specific genetic and epigenetic alterations and subsequently, multiple host and tumor interactions. In recent years, alterations on the immunosurveillance system have been pointed as critical on CRC progression and prognosis [2,3]. A correlation between tumor infiltrating lymphocytes (TILs) and earlier stage and aggressiveness has been described in CRC [4]. Several mechanisms of immunity modulation have been described, including interference with cytotoxic T cells (Tc) and natural killer (NK) cells, the main agents that destroy malignant cells through the perforin and granzyme-mediated apoptosis or by death receptor-ligand systems [5]. In addition to cytotoxicity, γδ T cells are able to phagocytose and present tumor antigens to CD8+ αβ Τ cells [6]. Likewise, γδ T cells are critical for a protective immune response against tumor through IFN- γ production. Murine experiments showed that γδ T cells were the first group of T cells recruited into tumor injection site [7,8]. Although, this cytokine is necessary in the protective responses against tumor development, its concentration may not be an indicative parameter of tumor response because both NK, NKT and gamma delta T cells might provide the early source of this cytokine [9,10]. Also, IL-22 produced by γδ T cells is a supervisor of the DNA damage response against environmental genotoxic factors in intestinal epithelial stem cells avoiding malignant transformation and cancer development [11].

Increases on T cell apoptosis has been identified both in peripheral blood and tumour samples, in different types of cancer [12–16]. In addition, the decrease in T cells in peripheral blood of patients with CRC has been linked to decreased survival and higher node-involvement [17–20]. Apoptosis of T cells also showed a significant correlation with Dukes stage, lymphatic and vascular metastasis, and patients age [21,22].

γδ T cells play a crucial role on the defence against infections and tumors, and they have been the subject of extensive cancer research [23,24]. The direct recognition of antigens without previous processing by the major histocompatibility complex together with the lack of alloreactivity make γδ T cells a natural target for tumour downregulation [25–27]. Regarding the subject of our work, one study has correlated lower circulating γδ T cell counts with worst survival [28]. However, the clinical relevance of circulating γδ T cells and their activated-apoptotic status in patients with CC has not been well defined.

The aim of this study was to measure the levels and differentiation status, incluing activation, of αβ and γδ T cell subsets and their apoptotic in peripheral blood samples of patients with newly diagnosed CC, without specific antitumor treatment, vs healthy subjects and the relation with patient characteristics.

## 2 Materials and methods

### 2.1 Subjects of study

In this prospective case-control study, a total of 96 patients with CC confirmed by histopathology were recruited, except for rectal cancer, vs. a control group of 48 healthy subjects matched for sex and age ± 2 years, one control for every two cases. Patients were diagnosed in our hospital in the last year, with criteria for surgical intervention.

Healthy subjects should include the following criteria: absence of inflammatory, autoimmune, acute infections or known immunodeficiency diseases; and no immunosuppressive or antibiotic treatment or any kind of vaccine during the six previous months. Patients with cancer should have the same criteria, in addition, they should not have received any specific treatment of their disease, chemotherapy, radiotherapy or immunological drugs.

The study was carried out according to the Declaration of Helsinki and was approved by the Ethics and Investigation Committee of the Arnau de Vilanova Univertity Hospital (Valencia, Spain). Written informed consent was obtained from all participants.

### 2.2 Methods of blood sample analysis

Blood cell counts were obtained before surgery.

### 2.3 Cell isolation for apoptosis evaluation

Peripheral blood mononuclear cells (PBMC) were isolated from EDTA anticoagulated blood by centrifugation on density gradient. Cells were then washed twice in phosphate buffered saline (PBS), and suspended in 0.5 ml of PBS/human serum albumin (1:1).

### 2.4 Apoptosis evaluation

The apoptosis detection was performed with ANNEXIN V-FITC/7-AAD Kit (Beckman Coulter, Inc, Indianapolis, IN), based on the binding properties of annexin A5 to phosphatidylserine and on the specificity of 7-amino-actinomycon D (7-AAD) for DNA guanine-cytosine base pair, without any variations of the protocol.

### 2.5 Functional analysis of γδ and αβ T cells

Flow cytometry was performed as previously described [29]. The following monoclonal antibodies were used: anti-TCR PAN αβ, anti-TCR PAN γδ, CD19, CD56, CD4, CD3, CD8, CD45, CD45RA and CD62L. The panel was designed in a way to avoid interferences between adjacent fluorescence, so that low-density populations could be detected. Monoclonal antibodies were conjugated with Phycoerythrin (PE), R-phycoerythrin-cyanine 7 (PC7), Alexa Fluor 750 APC (A750 APC), Alexa Fluor 700 APC (A700 APC), Pacific Blue (PB) and Krome Orange (KRO) (Beckman Coulter, Miami, Florida).

Acquisition and analysis were done on a Beckman-Coulter multiparameter flow cytometry analyzer, Navios (Miami, Florida) and later analyzed with Kaluza Software. A total of 100,000 events were acquired. Absolute counts of circulating cell subsets were calculated using the percentages obtained by flow cytometry, and the leukocyte count was obtained from the haematological analyser, using a dual-platform counting technology.

**Fig 1:**
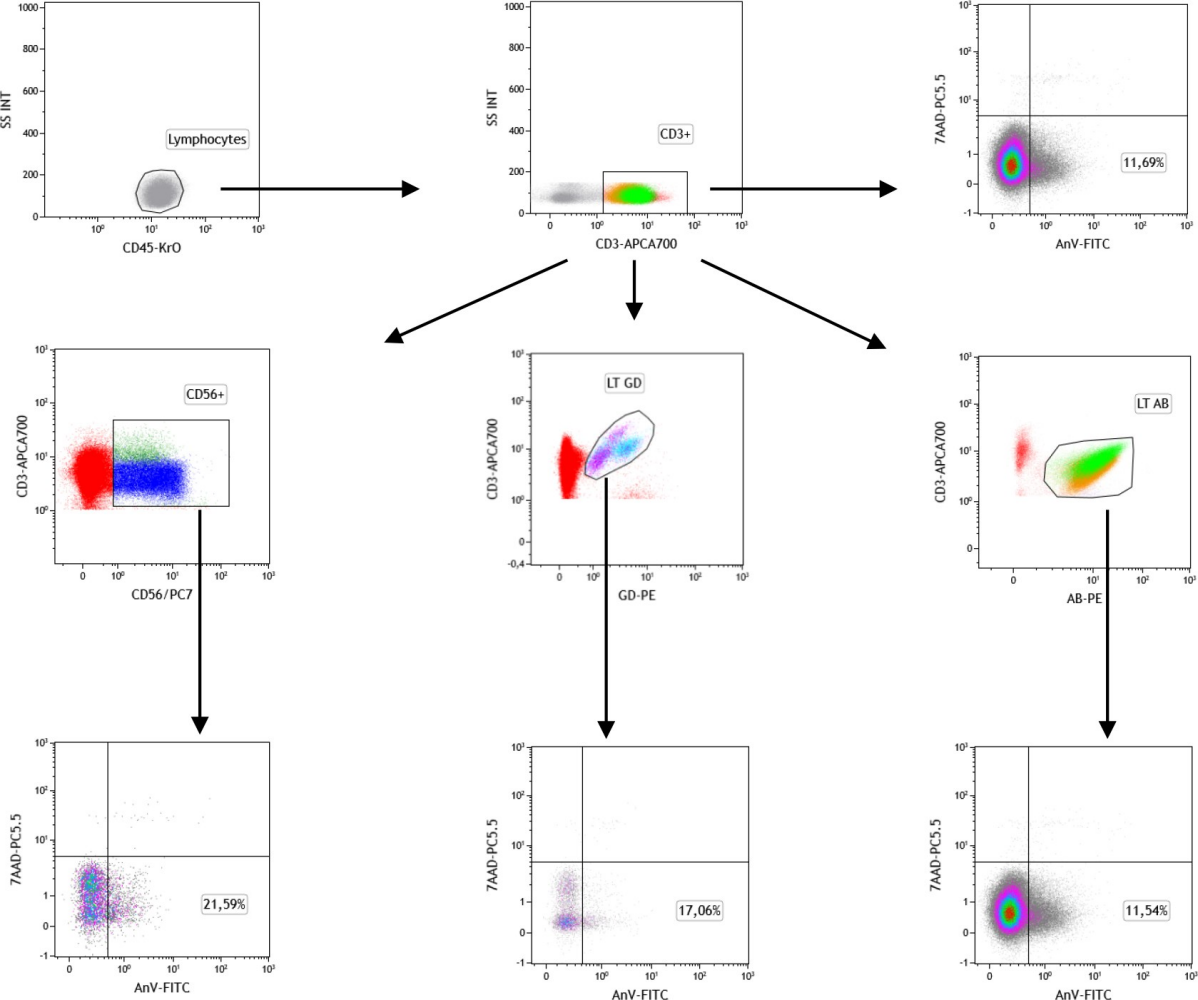
Gating strategy for T cell subsets. Peripheral blood events were measured against forward and side scatter parameters, and total lymphocytes were selected. Cells positive for CD3 were first selected. CD56+ αβ and γδ T cell subsets were analysed. Gating strategy for apoptosis (annexin V/7-AAD) was by using plots of CD3+, CD3+ αβ, CD3+ γδ, CD3+CD56+.

**Fig 2:**
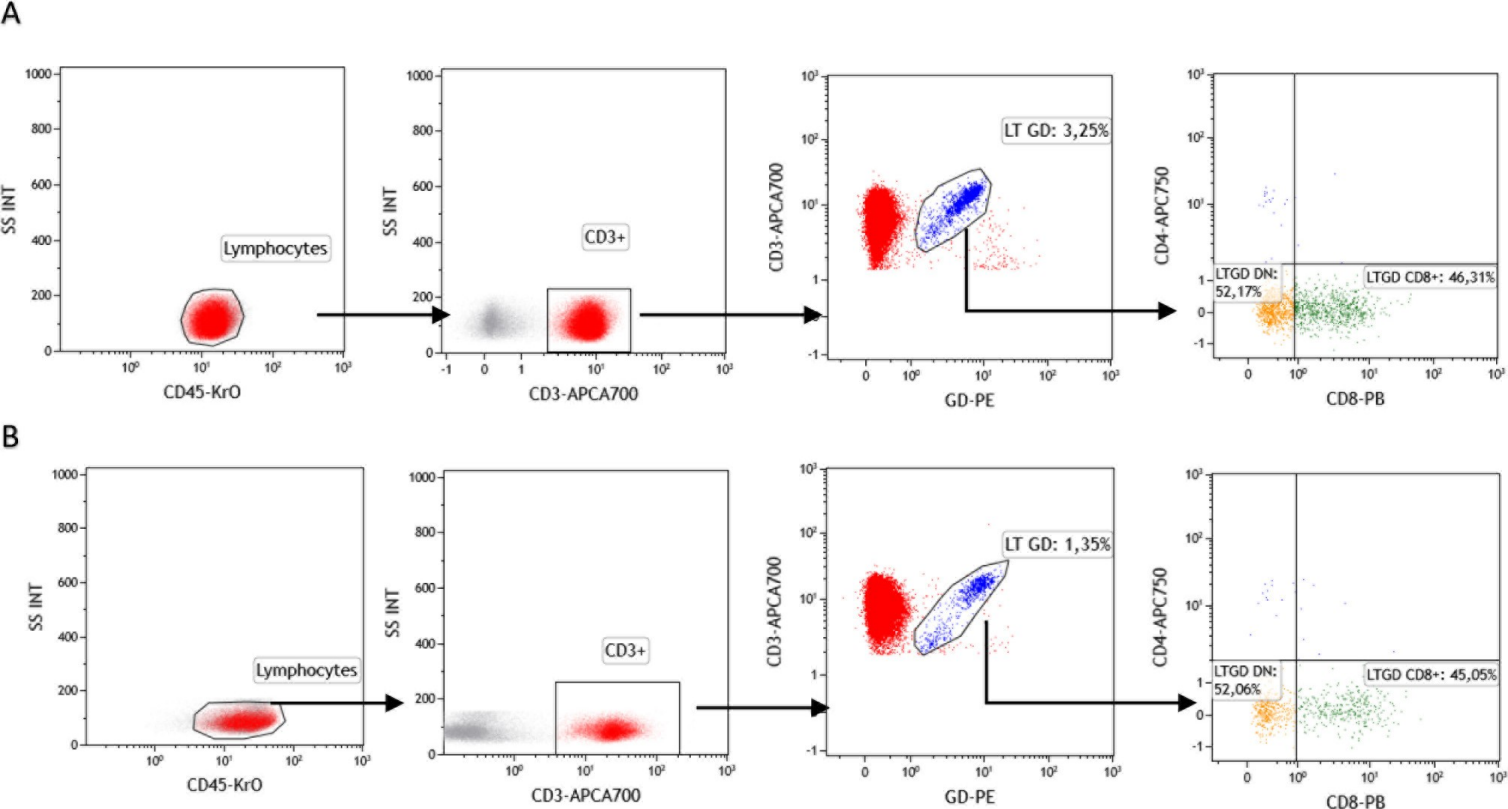
Representative plots of flow cytometric analysis using monoclonal antibodies anti-TCR PAN γδ, CD3, CD4, CD8. The squares represent peripheral blood γδ T lymphocytes and subsets, CD8 γδ T cell percentages (LTγδ CD8), and LTγδ Double Negative cell percentages (LTGD DN) for controls (A) and patients with colorectal cancer disease (B).

A range of internal quality assurance procedures were followed, including daily calibration of flow cytometer optical alignment and fluidic stability using Flow-Check fluorospheres and daily monitoring of whole-blood preparation procedures and monoclonal antibody reactivity using Immuno-Trol (Beckman Coulter, Miami, Florida) control cells. An external quality assurance procedure was also implemented through participation in a performance-monitoring network (Figs 1 and 2).

### 2.6 Measurement of ciculating cytokines (TNF-α and IFN-γ)

The levels of serum cytokines were analysed using LEGEND MAX™ Human TNF-α or IFN-γ ELISA Kits (BioLegend San Diego, CA) according to manufactured recommendations. The minimum detectable concentration of IFN-γ and TNF-α is 3.5 and 5.6 pg/ml, respectively. Serum samples were tested in duplicate.

### 2.7 Statistical method

To compare the quantitative variables mean values, Student *t* test was used if normality was assumed (Kolmogorov-Smirnov test). When normality was not accepted, the Mann-Whitney *U* test was used. ANOVA test was used to analyze the differences among group means. Correlation studies using Pearson Test were performed to compare T cell subsets frequency and apoptosis. *P* values < 0.05 were considered statistically significant. The data were analyzed using the Statistical Package for Social Sciences (SPSS 19.0; SPSS Inc., Chicago, IL).

## 3 Results

### 3.1 Patient characteristics

The characteristics of patients with cancer are shown in Table 1. A total of 144 subjects, 96 colon cancer patients and 48 controls were recruited. The mean age of cancer patients was 69.5 ± 11.5 years vs. 65.9 ± 10.4 years in the controls, p = 0.103. 60 male vs 36 females in cancer patients, vs 30 male and 18 female in healthy controls.

**Table 1:** Characteristics of patients with colon cancer (*n* = 96).

			*Media ± SD*
	***Media ± SD***	Analyzed lymph nodes	17.1 ± 8.1
Age	69.5 ± 11.5	Lymph nodes metastasis	1.3 ± 2.2
CEA (ng/ml)	8.3 ± 17.4		
CA 19–9 antigen (u/ml)	14.0 ± 14.9	Histologic type	***n* (%)**
Relapse time (months)	10.4 ± 6.6	*- Adenocarcinoma (AC)*	75 (78.1)
		*- Mucinous AC*	14 (14.6)
Gender	***n* (%)**	*- Infiltrant on adenoma AC*	6 (6.3)
*- Male*	60 (62.5)	*- Neuroendocrine AC*	1(1.0)
*- Female*	36 (37.5)	Grade	
TNM Staging		*-Low grade*	54 (56.3)
*- 0*	1 (1.1)	*-High grade*	42 (43.8)
*- I*	31 (32.3)	Invasion	
*- II*	27 (28.1)	*- Subserosa*	43 (44.8)
*- III*	33 (34.4)	*- Muscular*	28 (29.2)
*- IV*	4 (4.2)	*- Submucosa*	14 (14.6)
Recurrent	8 (8.4)	*- Serosa visceral*	5 (5.2)
Poliposis	60(62.5)	*- Neighboring organ*	5 (5.2)
Synchronous cancer	7 (7.3)	*- In situ*	1 (1.0)
		**Angiolymphatic Invasion**	40 (41.7)
Tissue	***Media ±SD***	Vascular invasion	7 (7.3)
*Tumor size (cm)*	3.8 ± 1.5	Lymphatic invasion	40 (41.7)
*Distance margins*	8.4 ± 7.5	Perineural invasion	6 (6.3)

*SD* standard deviation, *Histologic type* (OMS) SNOMED (Systematized Nomenclature of Medicine) code, *TNM* Tumor-Node-Metastasis, *CEA* carcinoembryonic antigen, *CA* carbohidrate antigen. Male 69.7 ± 11.3 vs female 69.2 ± 11.9, p = 0.850.

### 3.2 αβ and γδ T cells subsets and apoptosis of peripheral blood in colon cancer and healthy subjects

**Fig 3:**
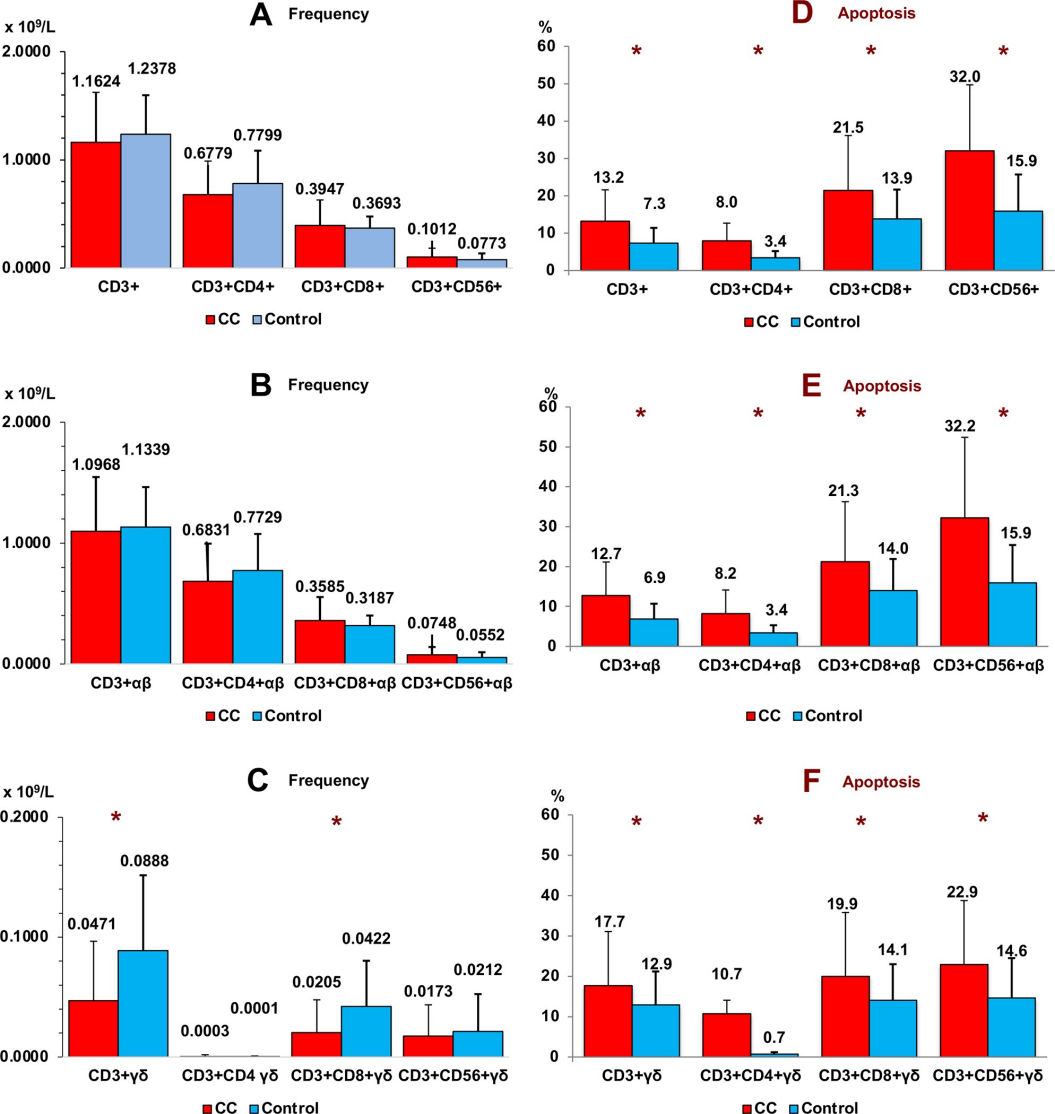
Frequency of T cell subsets. Frequency (panel A, B and C) and apoptosis (panel D, E and F) of conventional (panel A and D), αβ (panel B and E) and γδ (panel C and F) T cell subsets in peripheral blood of Colon Cancer (CC) and healthy subjects (control). Non parametric Test (Mann-Whitney U Test). (*p < 0.001). Results are expressed as mean ± SD.

We found a significant decrease in CD3+ γδ and CD3+CD8+ γδ T cells in peripheral blood of patients with CC vs. healthy subjects (p < 0.001). Apoptosis was significantly increased in all conventional T lymphocytes and both αβ and γδ T cell subsets in patients with CC vs healthy subjects (Fig 3). We did not find significant differences in relation to T cells subsets in cancer staging.

### 3.3 Correlations between frequency of αβ and γδ T cell subsets and apoptosis

In healthy patients apoptosis was positively correlated with the frequency of CD3+CD56+ γδ T cells (+0.413, p = 0.006). On the contrary, in CC patients a negative correlation was observed (-0.537, p < 0.001) (Pearson Test). No correlation was observed with the rest of the cell subsets.

### 3.4 Relationship of αβ and γδ T cell subsets and apoptosis with microvascular tumor infiltration in tissues

**Fig 4:**
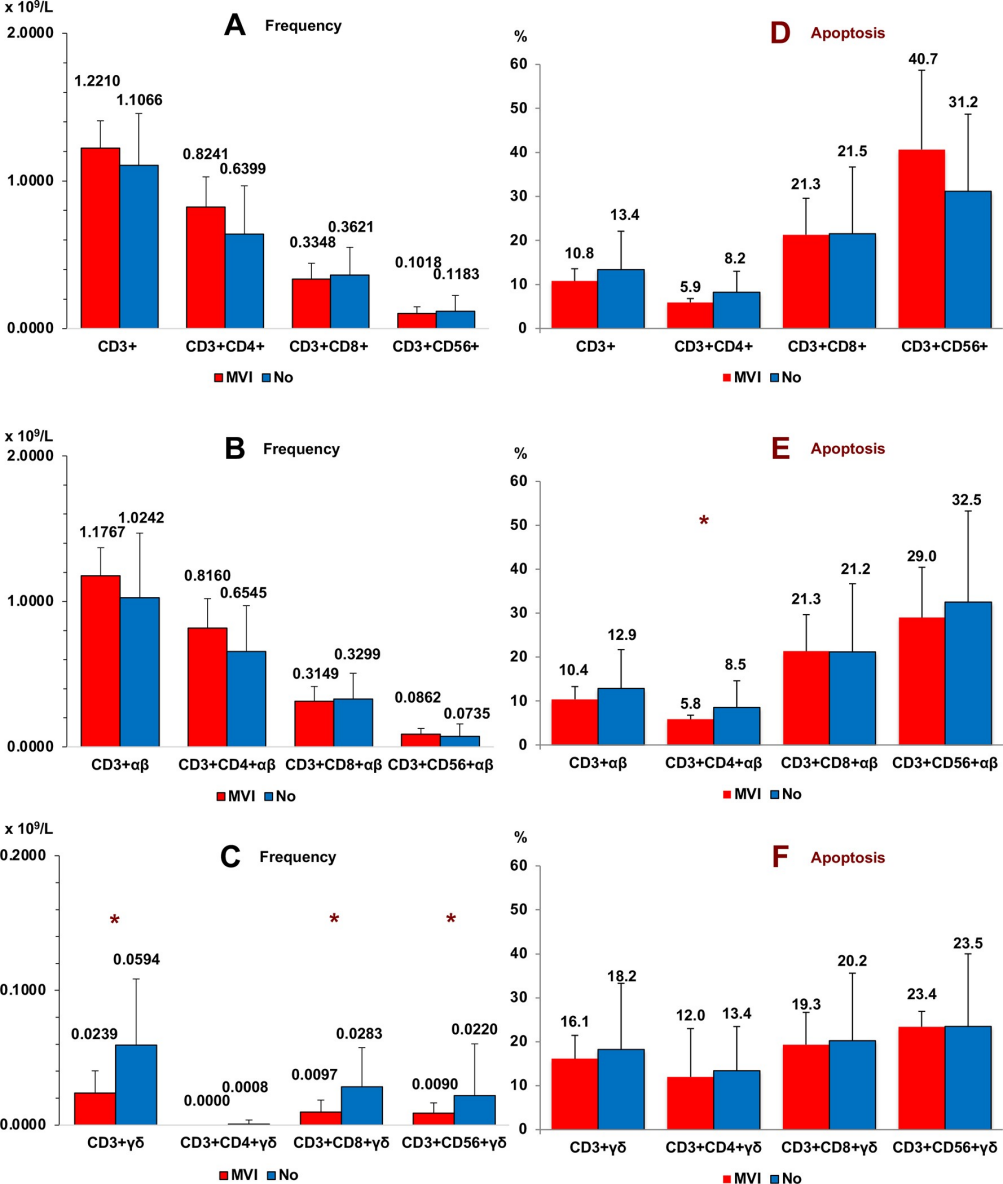
Microvascular tumor infiltration. Frequency (panel A, B and C) and apoptosis (panel D, E and F) of conventional (panel A and D), αβ (panel B and E) and γδ (panel C and F) T cell subsets in peripheral blood of Colon Cancer patients according to microvascular tumor infiltration (MVI) in tissues (*n* = 7). Non parametric Test (Mann-Whitney U Test). (*p < 0.05). Results are expressed as mean ± SD.

Fig 4 shows the frequencies of conventional (panel A), αβ (panel B) and γδ (panel C) T cell subsets, in peripheral blood of CC patients according to microvascular tumor infiltration (MVI) in tissues. Significant differences were only observed in the case of γδ T cells (CD8+ and CD56+). Thus, patients with vascular infiltration of tumor cells in their tissues had lower frequencies of γδ T cells in peripheral blood than patients without vascular infiltration. Apoptosis was slightly diminished in the CD3+CD4+ αβ subset while no changes were seen in the γδ T cells.

### 3.5 Relationship of αβ and γδ T cell subsets with a history of cancer (HC)

All T cell subsets were analysed in 15 patients with CC who had previously had cancer (CC+HC) (6 prostate, 5 colon, 1 breast, 1 endometrium, 1 leukemia, 1 insulinoma).

**Fig 5:**
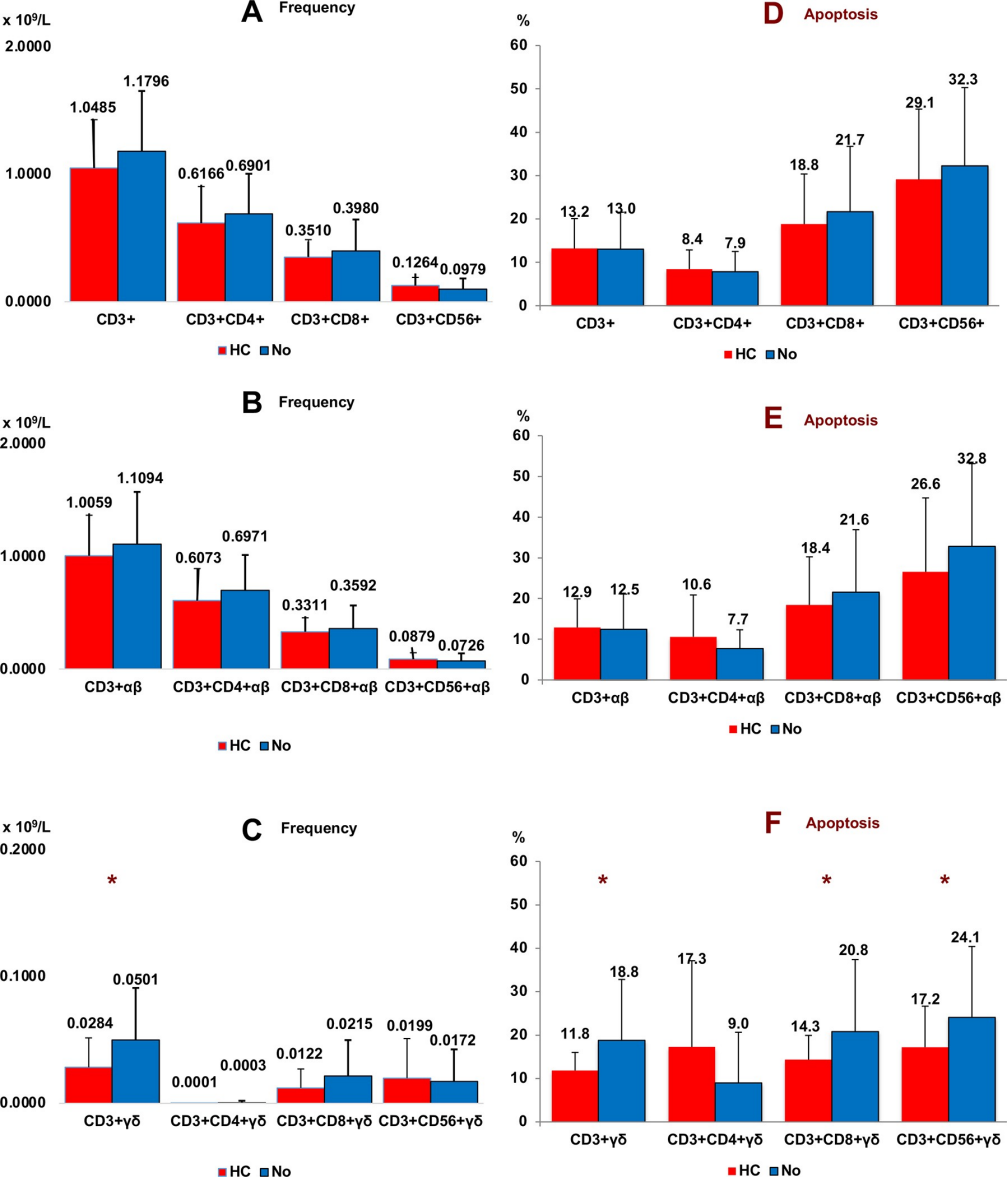
History of cancer. Frequency (panel A, B and C) and apoptosis (panel D, E and F) of conventional (panel A and D), αβ (panel B and E) and γδ (panel C and F) T cell subsets in peripheral blood of Colon Cancer patients according to History of Cancer (HC) (*n* = 15). Non parametric Test (Mann-Whitney U Test). (*p < 0.05). History of cancer: 6 prostate, 5 colon, 1 breast, 1 endometrium, 1 leukemia, 1 insulinoma. Results are expressed as mean ± SD.

The frequency of CD3+ γδ T cells was significantly lower in patients with CC and HC than observed in patients with CC who had no HC (Fig 5, panel C). CC+HC patients presented lower values of CD8+ γδ T cells although the difference was not significant (p = 0.114), probably due to the small number of samples, (*n* = 15). Keep in mind that 80% of peripheral blood CD3+ γδ T cells are CD3+CD4-CD8- γδ T cells (double negative). No differences were observed in the case of CD3+CD4+ or CD3+CD56+ γδ T cells. The average age in both groups was somewhat different, 74.8 ± 9.9 years in CC+HC vs 68.5 ± 11.5 years in CC patients without HC (p = 0.042).

The previous antitumor treatment received by HC was not the cause of the decrease in γδ T cells due to the time elapsed from the treatment to the current time (minimum three years).

Apoptosis was significant lower in γδ T cells from CC with HC patient’s vs CC patients without HC (CD3+ γδ, CD3+CD8+ γδ and CD3+CD56+ γδ) (Fig 3, Panel F).

### 3.6 Relationship of αβ and γδ T cell subsets with a synchronous cancer (SC)

**Fig 6:**
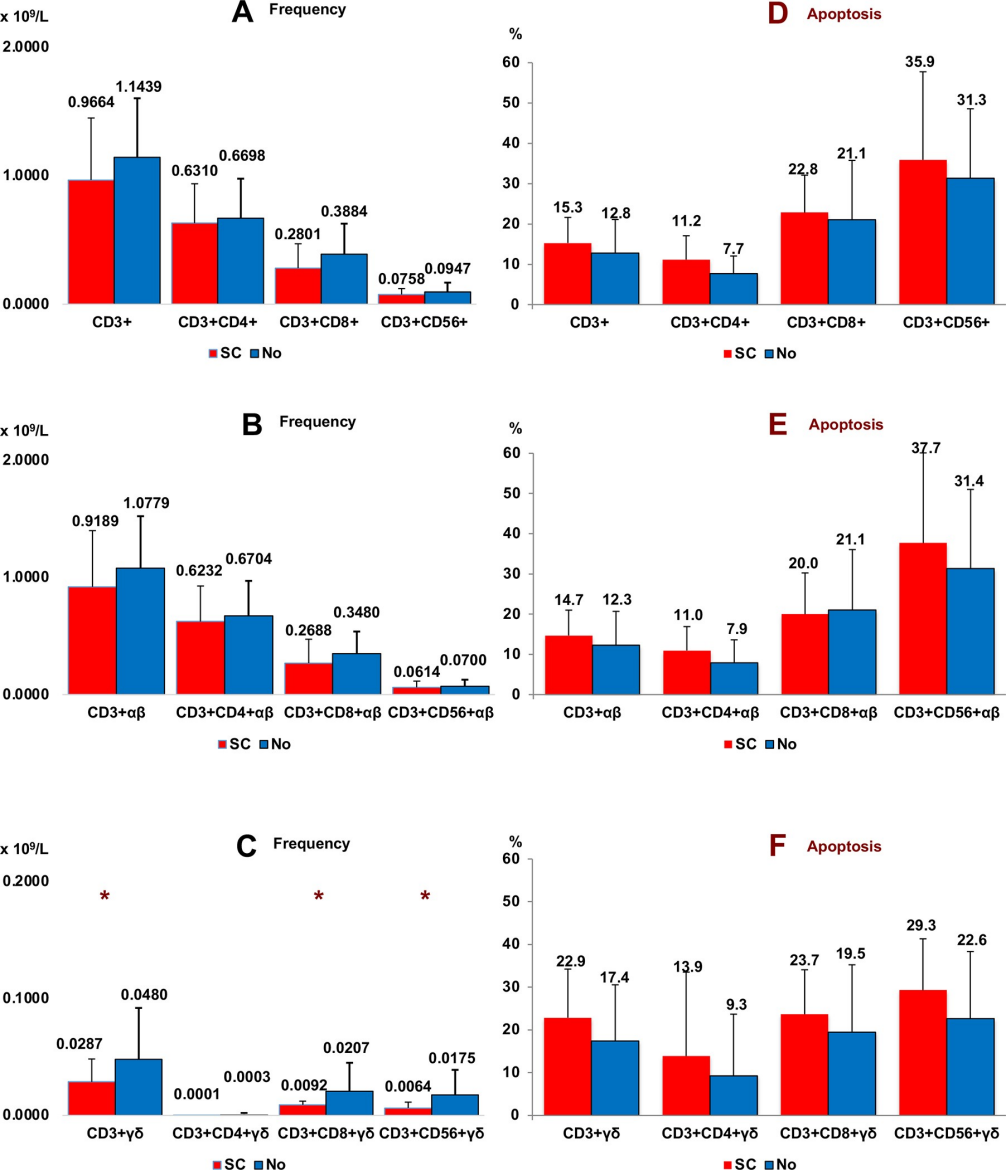
Synchronous cancer. Frequency (panel A, B and C) and apoptosis (panel D, E and F) of conventional (panel A and D), αβ (panel B and E) and γδ (panel C and F) T cell subsets in peripheral blood of Colon Cancer patients according to Synchronous Cancer (SC) (*n* = 7). Non parametric Test (Mann-Whitney U test). (*p < 0.05). Synchronous cancer: 5 colon, 1 breast, 1 endometrium. Results are expressed as mean ± SD.

Fig 6 shows the frequency of T cells in seven CC patients with a synchronous cancer (SC) (5 colon, 1 breast, 1 endometrium) compared with patients without a SC. Significant differences were observed between both groups with respect to γδ T cells (CD3+ γδ, CD3+CD8+ γδ and CD3+CD56+ γδ) (panel C). No significant differences were observed in the apoptosis of T cell subsets of CC patients according to the presence or absence of a SC. Of the seven patients with a synchronous cancer five were colon, one breast and one endometrium.

### 3.7 Natural killer cells (NK cells)

The frequency of NK cells was significantly higher in CC patients (0.4069 ± 0.2171 x10^9^/L) *vs* healthy control subjects (0.2964 ± 0.2149 x10^9^/L), p = 0.004. Similarly, apoptosis was also higher in subjects with CC (20.21 ± 15.1%) *vs* healthy individuals (6.0 ± 5.9), p < 0.001. There were no significant differences in patients with microvascular invasion, or a history of cancer or synchronous cancer.

### 3.8 T-cell differentiation stage and their apoptosis (percentage) of αβ and γδ cells

**Fig 7:**
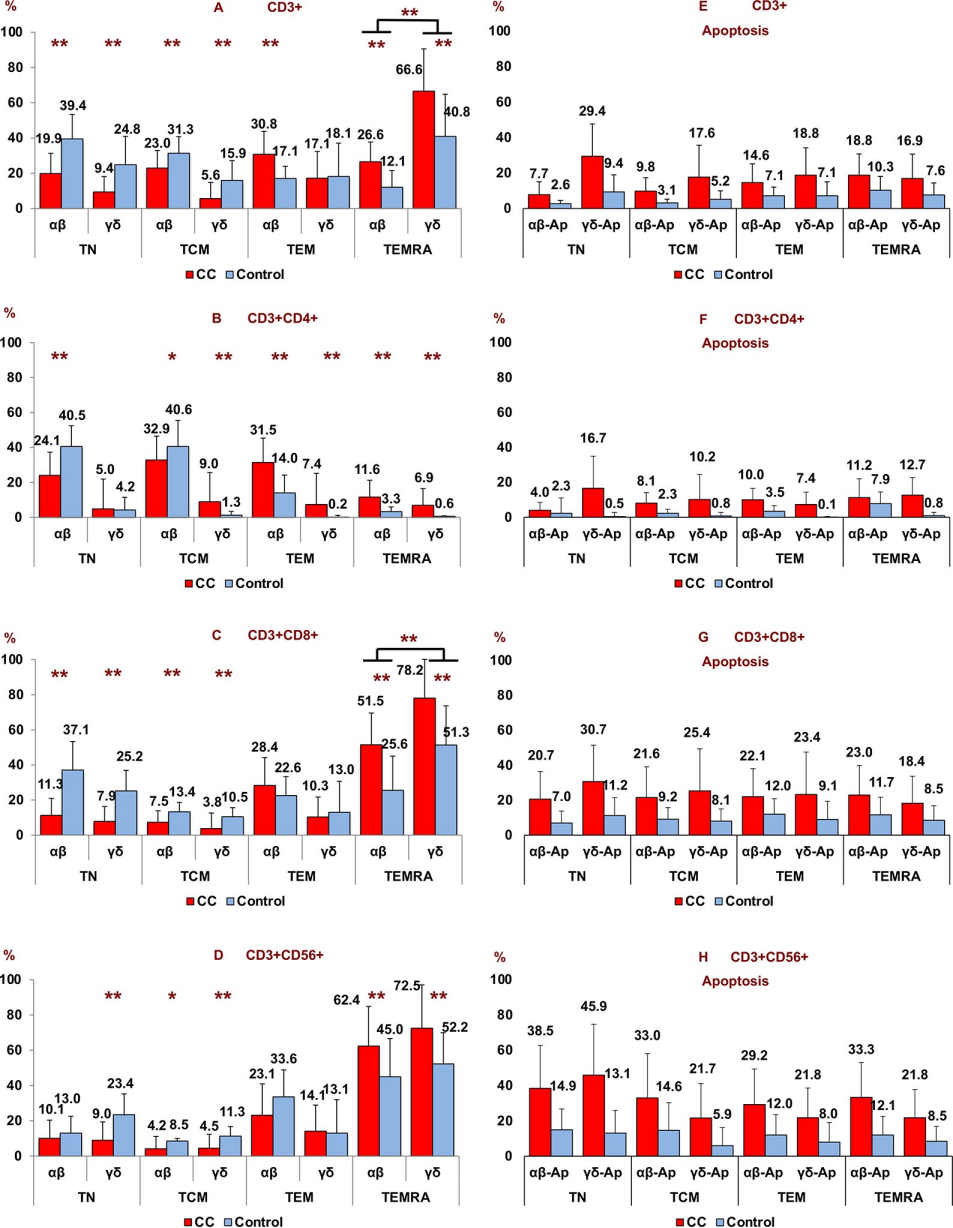
Percentages of T-cell differentiation state (panel A, B, C and D) and apoptosis (panel E, F, G and H) according to αβ and γδ T subsets (CD3+, CD3+CD4+, CD3+CD8+, CD3+CD56+) in peripheral blood of patients with colon cancer (CC) vs healthy subjects (HC). Non parametric Test (Mann-Whitney U Test). (*p < 0.05; **p < 0.001). Results are expressed as mean ± SD.

Patients with CC had a significant decrease in CD3+ αβ and γδ naïve T cells (TN), and central memory T cells (TCM) with respect to healthy subjects while CD3+ αβ effector memory T cells (TEM) were significantly increased in patients with CC *vs* healthy controls. CD3+ αβ and γδ terminally differentiated effector memory T cells (TEMRA) were significantly increased in patients with CC *vs* healthy controls (Fig 7A–7D). The biggest increase was registered in the case of γδ terminal effector memory T cells (TEMRA) where it had 66.6% (Fig 7A). This increase was due to CD3+CD8+ and CD3+CD56+ γδ terminal effector memory T cell (TEMRA) subsets. In these cases there was an increase of 78.2% (CD8+ γδ TEMRA) and 72.5% (CD56+ γδ TEMRA) (Fig 7C and 7D).

A significantly greater percentage of apoptotic cells were found in cancer patients in all T-cell differentiation stages compared to the control group (p < 0.001) (Fig 7E–7H).

### 3.9 Relation of personal and family history between CC patients and healthy subjets

Table 2 shows the difference of personal and family history between CC patients and healthy controls. We have found a significant decrease of γδ T cell frequencies in patients with CC who presented hyperlipidemia and cardiovascular disease. CD3+CD56+ γδ T cells at hyperlipidemia patients with CC, 0.0113 ± 0.0187 x10^9^/L *vs* 0.0188 ± 0.0267 x10^9^/L without hyperlipidemia, p = 0.015. CC patients with cardiovascular disease, CD3+CD56+ γδ T cells, 0.0164 ± 0.0220 x10^9^/L *vs* 0.0191 ± 0.0205 x10^9^/L without cardiovascular disease. CD3+ γδ T cells, at CC patients with cardiovascular disease, 0.0423 ± 0.0464 x10^9^/L *vs* 0.0529 ± 0.0430 x10^9^/L without cardiovascular disease. CD3+CD4+, 0.5649 ± 0.2487 at cardiovascular disease *vs* 0.7695 ± 0.3274 at no cardiovascular disease, p = 0.001, and CD3+CD4+ αβ T cells, 0.5634 ± 0.2449 *vs* 0.7802 ± 0.3292, p = 0.001. Body Mass Index at CC was: 26.6 ± 4.9 *vs* 25.2 ± 3.8 at healthy subjects, p = 0.148.

**Table 2:** Relation of personal and family history between CC patients and healthy subjects.

	CC (*n* = 96)	H (*n* = 48)		
	*n* (%)	*n* (%)	*OR (IC95%)*	*Sig*
Tobacco	31 (32.3)	21 (43.8)	0.7 (0.5–1.1)	NS
Alcohol	8 (8.3)	9 (18.8)	0.4 (0.2–1.1)	NS
Hypertension	56 (58.3)	13 (27.1)	2.2 (1.3–3.5)	< 0.001
Cardiovascular Disease	19 (23.8)	2 (4.2)	5.7 (1.4–23.4)	0.003
*-Ischaemic heart disease*	11 (15.1)	2 (4.2)	3.6 (0.8–15.6)	NS
*-Disease of arteries*	3 (12.0)	0 (0.0)	-	NS
*-Cerebrovascular*	8 (27.6)	0 (0.0)	-	NS
Diabetes Mellitus	18 (18.8)	3 (6.3)	3.0 (0.9–9.7)	0.048
Hyperlipidaemia	43 (44.8)	23 (47.9)	0.9 (0.6–1.4)	NS
COPS	10 (10.4)	6 (12.5)	0.8 (0.3–2.2)	NS
OSAHS	12 (12.5)	3 (6.3)	2.0 (0.6–6.8)	NS
Family Colon Cancer	14 (14.6)	4 (8.3)	1.8 (0.6–5.0)	NS
Family Cancer	21 (21.9)	17 (35.4)	0.6 (0.4–1.1)	NS
Obesity	6/28 (21.4)	5/48 (10.4)	2.1 (0.7–6.1)	NS

CC: Colon Cancer. H: Healthy. COPS: Chronic Obstructive Pulmonary Disease. OSAHS: Obstructive Sleep Apnea-Hypopnea Syndrome. NS: No Significant.

### 3.10 Tumor necrosis factor alfa (TNF-α) and interferon gamma (IFN-γ) in serum of CC patients

**Fig 8:**
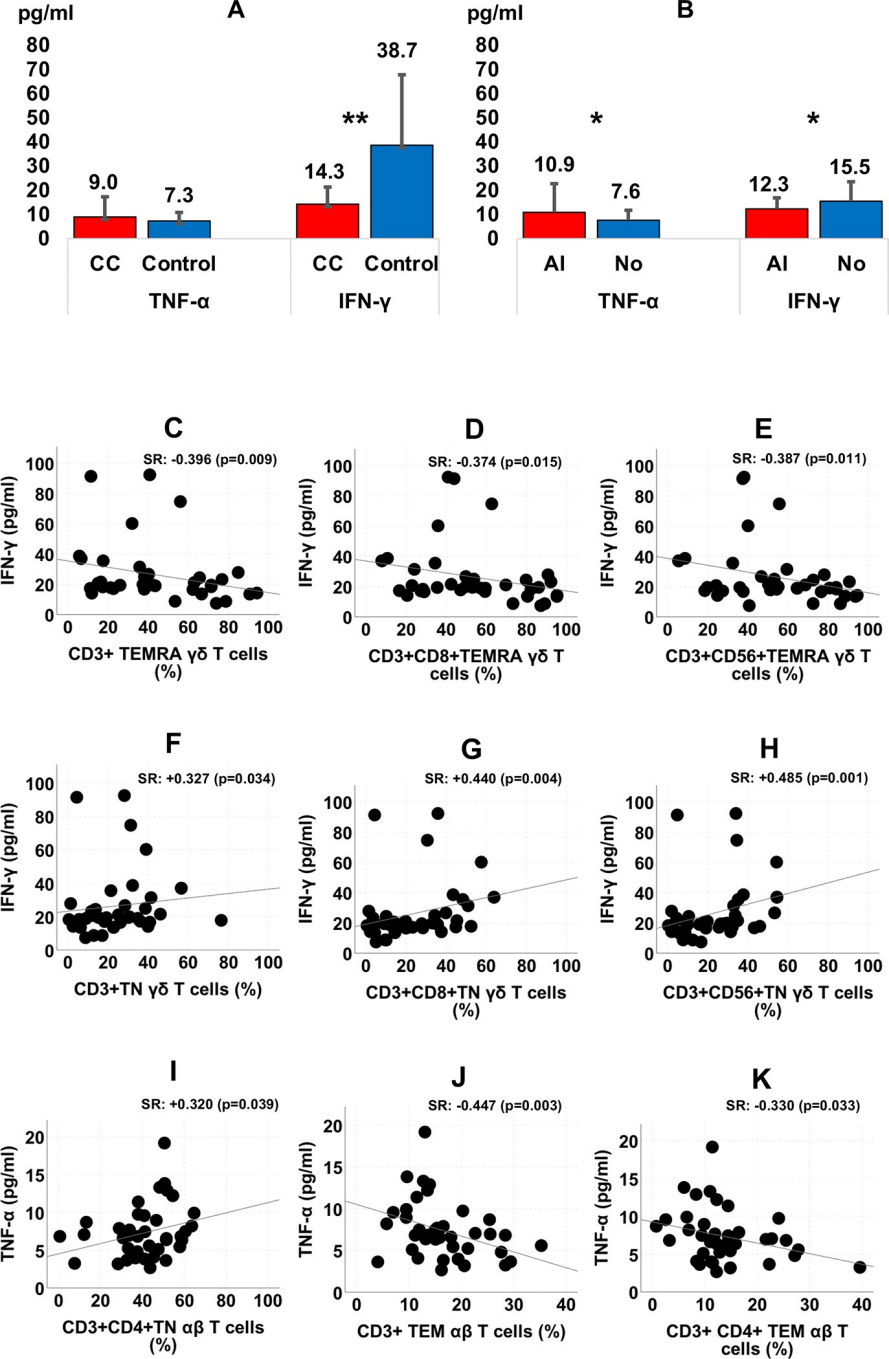
Differences of TNF-α and IFN-γ serum levels according to Colon Cancer (CC) (n = 96)/Control (n = 48) (panel A) and Angiolymphatic Invasion (AI) of CC patients (n = 40) (panel B); pg/ml: Picograms per milliliter. (*p < 0.05; **p < 0.001). Correlation of TNF-α and IFN-γ with T cell subsets in healthy subjects (panel C, D, E, F, G, H, I, J and K). SR: Spearman Rho correlation.

Serum levels of TNF-α and IFN-γ were analyzed. A very significant reduction of IFN-γ was observed in CC patients compared to healthy controls (p < 0.001). A reduction in IFN-γ levels was also observed in patients with angiolymphatic tissue invasion (p < 0.05) (Fig 8A and 8B).

In healthy controls, a positive correlation was observed between serum levels of TNF-α and IFN-γ and the different lymphocyte subpopulations studied. This correlation was not observed in CC patients (Fig 8C–8K).

## 4 Discussion

In this work we demonstrated for the first time a decrease of CD3+ γδ and CD3+CD8+ γδ T cells in peripheral blood of patients with newly diagnosed CC, compared with healthy subjects. In addition, we found an increase in apoptosis of all αβ and γδ T cell subsets in CC patients.

As mentioned above, low lymphocyte counts have been linked to poor prognosis in several cancer types including CC [14]. With respect to γδ T cells, one study has described a decrease of CD3+CD56+ γδ T cells in naïve nasopharyngeal carcinoma patients [30]. In another study including breast cancer patients, deficits in γδ T cell peripheral blood percentages were also observed [31]. Chen et al. demonstrated a significant decrease in the gene expression of γ, δ, ε and ζ chains in peripheral blood T cells of acute myeloid leukaemia patient’s vs healthy subjects [32].

No correlation is observed between apoptosis or γδ T cell levels in peripheral blood and tumour stage, even when stratified by earlier (stage I—II) or advanced (stage III—IV) (data not shown). The only factor that correlates with lower T cells counts was microvascular invasion (MVI). This situation has been attempted to relate to the possible haematogenous spread of tumor cells, and as an independent risk factor for poor prognosis in many solid tumors [33–38]. We showed a relationship between γδ T cell subsets in peripheral blood and MVI in tissues. Interestingly, this relationship did not appear in the case of the αβ T cells. This is of enormous importance, since it suggests the fundamental role of γδ T cells in avoiding the invasion of the tumour via the blood to other body locations and therefore, a fundamental role in avoiding metastasis [24].

A new subtype of γδ T cells with marked immunosuppressive phenotype in which IL-17 production predominates has recently been identified within the tumor microenvironment. These pro-tumor γδT17 cell subsets can express different phenotypes such as naïve-like, effector memory and Treg-like or Th2-like ones. The γδT17 cells promote tumor progression by inhibition of dendritic cell effector function and PD-1/PDL-1 immune checkpoint [39]. In mouse tumor models γδT17 cells promote tumor cell growth [24]. However, the small proportion of IL17-producing γδ T cells in peripheral blood could do their significance in the results of our study negligible. It has also been shown that γδT17 cells are the major cellular source of IL-17 in human colorectal cancer and γδT17 infiltration positively correlated with tumor stages and other clinicopathological features. Moreover, epithelial barrier failure caused by tumor might lead to commensal bacteria and bacterial product invasion. Consequently, a γδT17 de novo polarization is produced by dendritic cells activated by microbial products [40]. Therefore, it is not likely that the decrease of peripheral blood γδ T cells demonstrated in our study is due to their migration into cancer tissues.

It is of special interest that patients with CC who had previously cancer have less γδ T cells in peripheral blood than patients without a history of cancer. In our study, patients with CC had around 50% less γδ T cells than normal subjects. In addition, within the group of CC patients, the frequency of γδ T cells decreased by half in the HC subgroup. This fact shows that a history of cancer produces a 75% decrease in γδ T cells with respect to healthy subjects. This could suggest that these patients have a previous deficiency of these cells and a predisposition to cancer. A prospective cohort study could answer this question.

Synchronous colorectal cancer (SCC) refers to more than one primary colorectal carcinoma detected in a single patient at initial presentation. SCCs account for 5% of all colon cancers diagnosed and are associated with a decrease in survival compared to the rest of solitary tumors [41,42]. A metachronous colon cancer (MCC) is defined as a secondary colon cancer occurring more than six months after the primary tumor. The appearance of SCC and MCC is influenced by several individual and family factors. These include inflammatory bowel disease, especially Crohn's disease [43]. According to Maser et al. 35% of patients with Crohn and colon cancer undergoing surgical resection develop MCC. Likewise, 50% of patients with resection due to dysplasia present a new posterior dysplasia [44]. In our present study CC and SCC were associated with a decrease in γδ T cells. This decrease was also very evident in patients with CC and history of cancer. Previously, a deficiency of these cells on Crohn's disease was demonstrated by us [29]. Therefore, the low frequency of γδ T cells is a common element in these situations and suggests that this immunodeficiency could be related to both diseases, a fact that had previously been suggested in the case of Crohn's disease [45]. Among the cancer patients in our study, only two had a history of inflammatory bowel disease (one ulcerative colitis and another Crohn's disease). The antitumor treatment received by patients with a history of cancer could have influenced the decrease in T cells. However, this is very unlikely for the time elapsed from the treatment to the current time (years). In addition, this treatment would have caused a deficit of all T cell populations and in these patients only γδ T cells were diminished while αβ T subsets presented normal values.

T cell apoptosis is increased in peripheral blood and tissues of patients with different tumours patients. This fact was also proven in peripheral blood of the present study. The increase in apoptosis relates to all T cells, including αβ and γδ T cell subsets. In patients in whom a deficiency of γδ T cells was observed and higher rates of apoptosis, a negative correlation was observed between both parameters. This fact was especially evident in the case of CD3+CD56+ γδ cells. On the contrary, in healthy individuals the correlation was positive, the greater the number of CD3+CD56+ γδ cells, the greater the apoptosis. This means that in healthy individuals these cytotoxic cells do not receive the inhibitory signals of apoptosis expressed by tumor cells. According to Alexander et al. [46] the efficacy of tumor killing by T cells is related to their ability to resist apoptosis. In the case of CC patients, apoptosis is increased and as a consequence all T cells are diminished. This decrease results in a decrease in the γδ population and specifically in CD3+CD56+ γδ subset responsible for the immunosurveillance and destruction of cancer. However, a negative correlation was observed between CD3+CD56+ γδ levels and their apoptosis rates in CC patients. Alexander et al. observed that CD56+ γδ T cells are more resistant to FasL mediated apoptosis compared to CD56- γδ T cells suggesting that this relative apoptosis resistance could explain the enhanced anti-tumor activity of CD56+ γδ T cells. This means that, despite being diminished, these cytotoxic cells receive signals inhibiting apoptosis through the recognition of molecules present in cancer cells by binding to the activating NKG2D receptor expressed on CD56+ γδ T cells [47]. Nonetheless, the low number of CD3+CD56+ γδ cells in our CC patients could be insufficient to control tumor growth.

A linear model of T cell differentiation after antigenic stimulation from *naïve* T cells–TN- (CD45RA+ CD62L+ CCR7+) to central memory T cells–TCM- (CD45RA-CD62L+ CCR7+) has been suggested. These long-lived cells would colonize lymphoid tissues and, after recognizing the antigen again, they would lose the expression of CD62L that would facilitate their migration to peripheral tissues. In these locations they would become peripheral effector memory T cells -TEM- (CD45RA-CD62L-CCR7-) and terminal effector memory T cells -TEMRA- (CD45RA+CD62L- CCR7-) both short-lived populations. These last two have a rapid effector capacity after antigenic stimulation, secreting IFN-γ and TNF-α, TEM cells, and contain large amounts of granzyme and perforin, which gives them their cytotoxic property, TEMRA cells, mostly, γδ TEMRA T cells [48,49].

Interestingly, the highest percentage of cells, both in healthy subjects and in CC, were at the being even higher in patients with CC, which suggests that TEMRA effector T cells direct the immune defense against the tumor. Alexander et al. [46] showed that the peripheral blood CD56+ γδ T cells of healthy subjects have higher antitumor cytotoxic activity than CD56- γδ T cells on tumor cell lines of squamous cell carcinoma of the head and neck by means of perforin-granzyme pathway. Therefore, it would be interesting to deepen the possible therapeutic effect of this subset in adoptive immunotherapy.

In summary, the results of our work confirm a decrease in the prevalence and activity of γδ T cells in peripheral blood of patients with CC compared to healthy subjects. Our study does not establish whether this finding is a consequence of the tumor process or a predisposition. However, it is clear to us that this immune mechanism is linked to the development or progression of colon cancer. Previously it has already been reported that peripheral blood, at least in part, mirrors the gastric mucosa in terms of the immune response [50]. If the pattern observed in peripheral blood is a mirror of the colon tissue, where γδ T cells are predominant, a loss of function of the γδ T cells in the colic mucosa would generate an immunodeficiency environment that facilitates the tumour process. Our findings reinforce γδ T cell function repair as an attractive therapeutic target in CC. Patients with CC present a deep downregulation in the systemic T-cell immunity. These variations are evident through all tumor stages and suggest that a deficiency in γδ T cell populations could be preventing control of tumor progression. This fact prove the role of immunomodulation on CC carcinogenesis.

It should be noted that the production of IFN-γ was positively correlated with all cell subpopulations in the healthy controls. In contrast, there were no correlations in cancer patients. Furthermore, a notable decrease in this cytokine was observed in these patients, which was more pronounced within the angiolymphatic invasion group. IFN-γ is secreted by activated professional antigen-presenting cells, CD4+ and CD8+ T cells, NK, NKT, B and γδ T cells. This cytokine is capable of attacking the tumor through several mechanisms. IFN-γ improves the recognition of tumor cells by immune T cells, promotes apoptosis of tumor cells, prevents tumor angiogenesis, and induces innate and T cell-mediated antitumor immunity [51]. Tahir et al. [52] described a loss of IFN-γ production by invariant NK T cells in patients with advanced cancer. Low levels of iNKT cells and IFN-γ combination act as an independent predictor for recurrence and survival in hepatocellular carcinoma [53]. Conversely, ovarian cancer patients with high levels of IFN-γ expression had significantly longer progression-free and overall survival [54].

In our study, IFN-γ was significantly reduced in CC patients compared to controls. The low levels observed in the angiolymphatic invasion group revealed that the diminution of this cytokine preceded critical status. In other words, low plasma levels of IFN-γ could be associated with an increased risk of evolution to critical status in CC patients. This fact suggests that the disease severity and progression of CC is associated with a depletion or impairment of cellular sources of IFN-γ in cancer patients also related with their higher levels of apoptosis. Analysis of serum samples for patient multiple time points could be useful to clear this question.

In conclusion, we have identified a decrease of the total γδ T cells count and higher rates of apoptosis in peripheral blood of patients with CC compared to healthy subjects. This decrease is more pronounced in patients with microvascular invasion, or a history of cancer and synchronous cancer. IFN-γ was significantly reduced in CC patients compared to controls and cytotoxic effector γδ T cells TEMRA (CD8 and CD56) are the proportionally most abundant T cells in peripheral blood of CC patients.
